# Hospital and Institutional News

**Published:** 1918-04-13

**Authors:** 


### HOSPITAL AND INSTITUTIONAL NEWS.

### AMERICA'S GIFT.

Major William Endicott, American Red Cross Commissioner for Great Britain, has handed to Sir Robert Hudson, the treasurer of the British Red Cross Society, a cheque for £250,000 from the people of America to alleviate the suffering caused in the great battle in France. This magnificent gift, which will be of incalculable benefit at the present critical time, is a true indication of the sincerity of America's allegiance to the Allied cause, and of her eagerness to take the fullest part in the struggle. At a special meeting of the Finance Committee of the Joint Red Cross Societies the chairman, Sir Robert Hudson, seconded by Sir Herbert Jekyll, moved a resolution, subsequently carried unanimously, assuring the American Red Cross officials that the close co-operation and cordial relationship between the Red Cross organisations of the two countries formed one of the happiest incidents of the work of mercy which the war had devolved upon them. At the same time a telegram was sent to the Commissioner for France and Belgium that the Society would gladly meet any extra demands that might be made upon them during the present crisis.

### THE ROYAL FREE HOSPITAL.

One of the most significant developments in London is surely that of the Royal Free Hospital. Ever since the great legacies which accrued to it a few years before the war, scheme after scheme has been planned; the case of King Edward VII.'s Hospital, Cardiff, is the most obvious recent parallel. With considerable funds, and an extended site, it is therefore hardly surprising to hear Mr. Alfred Langton state that they hoped the number of beds would be doubled. Women's wards, maternity beds, and children's wards are desired. These, no doubt, will add to the advantages of the women's medical school, which, with its 440 students, is now the biggest in London. With the impetus which the war has given to the demand for women doctors, the school should continue to grow; and though competition for hospital posts will be keener after the war, the woman doctor has been tried and not found wanting, and modern legislation is creating new posts for her in many directions. Therefore, an ambitious policy for the Royal Free Hospital and for its medical school seems to be the right one.

### THE FUTURE OF GOLDERS GREEN.

There is a good deal of obscurity as to the position as regards the Home of Recovery at Golders Green. In a speech at Worcester about three weeks ago the Minister of Pensions spoke of the undesirability of retaining the neurasthenic pensioners in the air-raid area, and suggested that, after all, it might be better to mix them with the "cheery chaps" to be found in a non-specialised institution. Now, Mr. Hodge, in speaking on medical matters, can only be, unless he is not the canny Scot we take him for, the mouthpiece of a medical opinion somewhere in the background. Yet the treatment of neurasthenic patients in separate hospitals or homes is apparently still the policy of the Ministry of Pensions, for we observe that at the annual meeting of the National Hospital for the Paralysed and Epileptic it was announced that the board had undertaken for the Ministry a Home of Recovery for these cases situated about twenty miles from London. This is consistent with the air-raid plan, but is not in accordance with a system of non-segregation. In respect of air raids it is notorious that the hospital patient in London, whether neurasthenic or not, stands them as well as the man in the street, if not better, having regard to respective physical conditions. Then the separation of neurasthenics from "cheery chaps" has not proved a failure, as is shown by the success of Lord Knutsford's hospital for shell-shocked officers and the record of Golders Green as disclosed in the letter to the *Times* from Mr. Drummond, the chairman of the Hospital for Epilepsy and Paralysis, Maida Vale, the hospital managing the first Home of Recovery. But the doctors are not unanimous as to separation, and "who shall decide when doctors disagree and soundest casuists, like you and me?" The future of the Golders Green Home of Recovery appears to be in the melting-pot.

### SEGREGATION FOR NERVE CASES.

In these circumstances we record our opinion that patients suffering from shell-shock, or from neurasthenia from other causes, are better treated in a special hospital by themselves. Apart from their own very decided opinion, which it is impossible to ignore, there are sound medical reasons for taking this course. Lest the opinion of the patients themselves should be lost or forgotten, we reproduce here the actual words of the resolutions that they passed among themselves, without any prompting or stimulation from outside their own ranks: (1) "That we, the undersigned patients at 'The Home of Recovery,' Golders Green, having benefited, and in many cases recovered, desire to express our contentment and satisfaction with remaining in this institution, having the utmost confidence in the secretary, the medical officers, and the conditions generally of the hospital. Our past experiences in general hospitals have taught us to appreciate being separated from other cases, especially from the so-called 'cheery man.'" (2) "That all patients are agreed that after their experience of other hospitals and the company of the so-called 'cheery chap' they prefer to risk the air-raid results before that of going to another hospital." When a mode of treatment can show such results as the following, there is little justification for changing it for a mode to which the patients emphatically object, and which rests, it appears, only on the recommendation of an amateur. Of the 186 patients discharged during the last five months 53 returned to their former employers by arrangement with the home, 33 went to new work found for them, 14 went to training in some skilled occupation, and 39 left "fit for work" and preferring to make their own arrangements, which they have not reported. Included in this number of 186 were 35 patients who were either medically unsuitable or who were not treated to a conclusion—that is to say, 66 per cent. of the men who were treated to a conclusion returned to work, and a further 24 per cent., making 90 per cent. in all, were rendered "fit for work."

### THE R.A.M.C. IN THE GREAT BATTLE.

From the current number of the *British Medical Journal* we learn something of the work done by the Medical Services during the brief German advance. The large numbers of wounded constantly arriving from more advanced positions made the evacuation of the casualty clearing stations a matter of the greatest difficulty, and the losses to the R.A.M.C. both in stores and *personnel* must certainly have been heavy. As far as possible, the nurses and sisters were sent out of danger at an early stage; but in all cases this could not be done, and not a few lost all they possessed in the disorder of a hasty departure. The immense total of prisoners claimed by the Germans must include very large numbers of wounded, who were unavoidably left behind upon the field of battle. A tribute is paid to the splendid behaviour of all concerned. Some units were unable to close up until the advancing Germans were practically upon them, but all ranks seem to have behaved with signal gallantry and devotion.

### CONVALESCENTS IN BILLETS.

The plan already adopted at West Bridgford of asking householders to provide beds for convalescent soldiers is one that may be followed elsewhere. Its object is to release beds for new cases earlier than would otherwise be the case. But the expedient of billeting convalescents has not much to recommend it, as previous experience has shown. Arrangements which were made with householders in the neighbourhood of the Manchester military hospitals to receive convalescents broke down because the householders could not procure the required rations from their local shopkeepers. Under the latest attempt to arrange this billeting convalescent soldiers will take their meals at the hospitals from which they come. Householders will have to provide clean beds and bedding for 6d. per night, and the military will be responsible for the washing. This scheme, which is mainly anticipatory, seems to us to be full of objections. First, billets in private houses are not the right place for convalescents. Secondly, the plan will severely tax the kitchens and dining-rooms of the hospitals. The second point is important; the first is vital. If the number of hospital beds has to be increased, the proper thing to do is to add directly to their number or to relieve them by increasing the number of convalescent homes.

### THE AMERICAN AMBULANCE TRAIN.

A large number of persons, including many American citizens, availed themselves of the opportunity of inspecting an admirably appointed ambulance train, built by the Great Eastern Railway Company at their Stratford works for the use of the United States Army in France, which was on view last week at Liverpool Street Station. The train contains all the latest improvements for the treatment and comfort of the sick and wounded. It consists of sixteen cars, is 912 feet long, and its weight, unloaded, apart from the engine, is about 442 tons. There is accommodation for 360 cot cases, three medical officers, three nursing sisters, forty-six staff and *personnel*, thirty-eight N.C.O.s and men, and two railway guards. There are nine ward cars, a pharmacy car, a staff car, a *personnel* car, two kitchen cars, and two brake cars, in one of which are separate wards for infectious cases. The train is vestibuled, and is fitted with electric light and eighty fans. On the inspection days a charge was made for admission, and the money received will be given to the fund for providing comforts for the Railway Engineering Section serving with the Army in France. A number of opportunities for inspection have been arranged in different localities before the train is sent abroad.

### DEATHS IN COTTAGE HOSPITALS.

Some misapprehension and misgiving appear to have been caused among the supporters of the Buxton and District Cottage Hospital in consequence of the deaths which have occurred. In dealing with this at the recent annual meeting, Mr. C. A. Bailey, who has acted as chairman during the past year, reviewed the hospital's record in this respect. He stated that out of the 988 cases which had been treated since the hospital started six years ago there had been 88 deaths. Mr. Bailey is reported to have added that as the average per year was only 7, and the percentage less than 4, it was very small. It is not clear how this average and this percentage have been arrived at. In one or more years the percentage must surely have been higher than 4, if of 988 patients treated 88 have died; since on the total the percentage is, roughly, 9. The hospital is not necessarily to blame for this; but a fuller explanation is desirable, and the reasons for the "bad" year or years should be made plain.

### HOSPITAL ACTIVITY IN BELFAST.

Since the war began the two wards which were not in use before have been opened at the Royal Victoria Hospital, Belfast, and it is hoped that this will mean that they will never be closed again. The need is twofold. First for the waiting patients and, secondly, for the medical school, which is now being attended by a larger number of students than at any time during the past ten years. The hospital claims to have been a model for a certain famous war hospital in France. Though the cost per bed has risen from £64 (in 1914) to £90, the hospital ended its financial year with a good balance in hand. General subscriptions nearly doubled, and those of the working men rose from £5,000 to £8,000. The Working Men's Committee and the Financial Aid Committee both deserve praise for their share in this success, for, as Mr. W. G. Anderson lately pointed out, the success of collections depends on organisation. Taking his estimate that there are 90,000 munition workers in Belfast, over £14,000 could be raised from weekly collections alone. Munition works will not last for ever, but since they provide hospitals with many patients and are often factories which will revert to their original manufacture after the war, now is the time to create an organisation among them which will survive the change to peaceful manufacture.

### THE COLLEGE OF AMBULANCE.

The future of the College of Ambulance as a school of instruction was considered at a recent meeting held at the institution, 3 Vere Street, Oxford Street, when Sir James Reid was in the chair. He urged that the College, which had been invaluable in training men and women in first-aid, should be permanently established as a national institution. The principal and founder, Sir James Cantlie, stated that 20,000 students had passed through the College, and that attendances at the daily classes numbered 500 a week. He also referred to the models and the elaborate museum illustrating field ambulance work and to the *x*-ray pictures of injuries, the concentration of which in one central building was a great advantage. Sir Malcolm Morris said that the best recognition of the services of ambulance men and women would be the establishment of a permanent training-school. For this £30,000 would be necessary. Such an institution for the benefit of civilians would be "on all-fours" with what the Royal Army Medical Corps had accomplished for the soldiers.

### DEATH OF A FORMER HOSPITAL SECRETARY.

A notable figure in hospital administration in Liverpool has passed away in the person of Mr. Joseph Unsworth, who was for forty-five years secretary of the Liverpool Medical Institution, later known as the David Lewis Northern Hospital. Mr. Unsworth's connection with the hospital extended from 1869 until his retirement in June, 1913, when his long service was recognised by the presentation of an illuminated testimonial, a substantial cheque, and a retiring allowance. In the work of obtaining funds for the rebuilding of the institution, Mr. Unsworth's efforts had much success. His forty-five years' service are claimed in Liverpool as a record. Mr. Unsworth's death took place at Port St. Erin, Isle of Man, where he had lived since his retirement. He was seventy-four years of age. Mr. Unsworth's original occupation was that of a schoolmaster.

### THE SPREAD OF TRENCH FEVER.

That frequent source of the diagnosis P.U.O. (pyrexia of unknown origin)—trench fever—thanks to the researches of a committee of investigators from the British and American Expeditionary Forces, is to have its own specific bacterium, though the organism has not yet been isolated. These workers have shown that the disease is spread by the body louse. The immediate effect of their discovery will be the erection of better laundry and bathing accommodation in the rear of the trenches. The personal hygiene of troops will be insisted upon more than ever. Incredible as it seems, trench fever and scabies have been responsible during the past three years for more sickness among the forces than any other two diseases. The importance of these researches can hardly be overestimated.

### THE RED CROSS IN HOLLAND.

The arrangements for the education of prisoners of war interned in Holland, which were commenced some months ago under the direction of Lord Sandwich, have now reached a stage where they are likely to be of high utility to the officers and men forced to spend a long period of inactivity in a neutral country. For the officers there are classes in French, Dutch, Russian, and even German, whilst shorthand is among the items of a highly practical curriculum. The Municipality of The Hague has generously let a part of the Fair Ground at Scheveningen to the British Red Cross at a nominal rent, and upon this a carpentry hut has been erected, which at present accommodates between forty and fifty men, but is capable of considerable expansion as the need arises. For learning artificial limb making a number of men have been taken into the workshops of Dr. Pohl at The Hague, a very important step, since there is sure to be a wide demand for artificial limbs after the war. Provision for hospital treatment includes the splendid mansion of the Baroness de Brienen at Clingendael, for the use of officers, and a Red Cross hospital with British doctors and nursing staff, which will be used till the Dutch authorities have completed their arrangements. The number officially notified as having been transferred is at present between 600 and 700 N.C.O.s and men and 120 officers, and 400 civilians are expected to arrive shortly from Germany.

### COMPLAINTS AGAINST A MEDICAL BOARD.

A satisfactory solution to the complaints made by the Lambeth Tribunal as to the composition of one of the Medical Boards at Camberwell, which was previously known as the No. 1 Travelling Medical Board of Battersea, referred to in last week's issue of The Hospital, has been received from the Ministry of National Service. In a letter to the clerk to the Tribunal, the Ministry states that the chairman of the Travelling Board, who, it was alleged, had been transferred to a similar position at Camberwell, was no longer at Camberwell. "The extract from the chairman's letter has been misunderstood," the letter went on to say. "He did not shirk the responsibility of rejecting men who were unfit or unlikely to become fit for any form of military service." The concluding portion of the letter from the Ministry of National Service should remove some of the doubts as to the fairness of the new medical examinations. "The

Commissioner of Medical Service for the London region," it states, "has arranged that no case referred by a tribunal for re-examination shall be placed before the National Service Medical Board that made the classification against which the appeal was pending. This will, it is hoped, go far to remove any cause for complaint." In a number of cases which have come before Tribunals since the National Service Medical Boards were set up more than 75 per cent. of the men have been placed in lower medical grades. There are few complaints now as to the perfunctory way in which the examinations are carried out.

### SOME ST. DUNSTAN'S SURPRISES.

A paper edited by Sir Arthur Pearson raises expectations, and certainly the *St. Dunstan's Review* is one of the most interesting papers we have read. It is full of unusual information and remarkable details, which describe at first hand how the blind may gain without the optic nerve many of the advantages of sight. Mr. Percy Way, the distinguished blind masseur, in a capital article in the March number on "Getting About Alone," says: "We depend on the extraordinary sensitiveness of the nerves of the face. . . . The natural sensitiveness of these nerves can easily be demonstrated by holding an object a few inches in front of the face of a totally blind person without previously warning him. He instantly becomes aware of its presence, though it may make absolutely no sound. By the exercise and cultivation of this sense a man can walk parallel to a wall or fence at a distance of several feet, becoming aware of the fact if he for a moment lessens or increases the distance between himself and it. . . . Soon this becomes a matter of second nature." Those, too, who were not born blind develop a mental sight which enables them to visualise rooms and places they have explored, while the sense of smell helps a man to distinguish one shop in a street from another. This is one only out of several excellent articles. The Notes by the chief are peculiarly interesting, including that on a new policy of certain insurance companies, which will now accept blind persons at ordinary rates for life and even accident insurance. The news from those now at work in the world is most interesting too. How various are their occupations? Private Albert Law, for instance, has now started in practice as a masseur. It should be added that his success began as soon as he gave up calling upon doctors with a guide. In finding his way to their houses alone he gave an earnest of his ability.

### THE MARCH "SEARCHLIGHT."

It is pleasant to see in the March number of the gazette of the 2nd Western General Hospital, Manchester, which is known as the *Searchlight*, a sketch portrait of the editor, Sergeant A. Wilson Hildreth, whose Notes, signed "Kappa," on books and persons have become a familiar feature of the paper. There is a curious article on "Visiting Day," in which the writer gives his (or her) impressions of the various patients visited. There is a slightly superior air in the criticisms which may, or may not, amuse those who can recognise the subjects of them under the disguise of their nicknames or initials. Book reviews and quotations from well-known authors help to fill the pages, and Arnold Warden provides several pen-and-ink drawings. The contributions from men overseas, who have formerly been patients, are continued, and help to vary the home and local news.

### SACCHARIN AND THE FOOD CONTROLLER.

It is satisfactory to know that the authorities anticipate that there will shortly be sufficient British-made saccharin to meet the heavy demand. Hitherto this country has had to depend very largely upon imported saccharin and the public has had to pay through the nose for this luxury. Until sugar supplies became reduced saccharin was regarded more as a drug than anything else; it was not, of course, a drug, but since it was prescribed for the gouty and the obese, and bought at a chemist's shop, the layman looked on it as a pleasant sort of medicine. But nowadays a saccharin tablet takes the place of a lump of sugar in our teashops, and consequently the demand for it has increased enormously, with the result that prices have been run up to extraordinary figures yielding colossal profits to the dealers. Now that a sufficiency of home-made saccharin is in sight the sugar department of the Food Control Office has very properly fixed maximum selling prices for tablets made from British saccharin; not only will those who use saccharin because they cannot get enough sugar benefit by this new arrangement, but the medical man who prescribes saccharin for his patients will have the satisfaction of knowing that they will be able to get it at a reasonable price.

### THIS WEEK'S DRUG MARKET.

The upward tendency of prices continues, and supplies of many commonly prescribed drugs are steadily dwindling. Bromides are difficult to obtain in quantity, and a further rise in quotations would seem almost inevitable. Borax and boric acid are both dearer. Methyl salicylate is almost unobtainable, tartaric acid and cream of tartar are very scarce, and prices continue to tend upwards. An inspection of the stocks of quinine in the London warehouses reveals the fact that the quantity is much larger than had been estimated; this is fortunate, but prices are hardly likely to decline, since even the larger stocks are small compared with normal times. Salicylates are unchanged in price, but aspirin has an easier tendency, and the output from the British factories appears to be increasing. Phenacetin is also in better supply. Antifebrin and hexamine are again rather dearer. Olive oil, almond oil, and medicinal castor oil are very scarce. It seems probable that there will be a further advance in the price of camphor, owing to the difficulty of obtaining supplies. Cloves are dearer, and clove oil is scarcer with an upward price tendency. Aloin and terpineol are both rather dearer. Chaulmoogra oil and oil of amber (oleum succini) are quoted higher. The scarcity of milk sugar is unrelieved. Isinglass has been sold at lower rates.

